# Mesenchymal stem cells loaded on 3D-printed gradient poly(ε-caprolactone)/methacrylated alginate composite scaffolds for cartilage tissue engineering

**DOI:** 10.1093/rb/rbab019

**Published:** 2021-05-16

**Authors:** Yanyan Cao, Peng Cheng, Shengbo Sang, Chuan Xiang, Yang An, Xiaochun Wei, Zhizhong Shen, Yixia Zhang, Pengcui Li

**Affiliations:** 1 Key Lab of Advanced Transducers and Intelligent Control System of the Ministry of Education, MicroNano System Research Center, College of Information and Computer, Taiyuan University of Technology, Taiyuan 030024, China; 2 College of Information Science and Engineering, Hebei North University, Zhangjiakou 075000, China; 3 Shanxi Key Laboratory of Bone and Soft Tissue Injury Repair, Department of Orthopedics, The Second Hospital of Shanxi Medical University, Taiyuan 030001, China; 4 Department of Plastic Surgery, Peking University Third Hospital, Beijing 100191, China; 5 Department of Biomedical Engineering, College of Biomedical Engineering, Taiyuan University of Technology, Taiyuan 030024, China

**Keywords:** three-dimensional printing, cartilage tissue engineering, PCL, methacrylated alginate, bone marrow mesenchymal stem cells

## Abstract

Cartilage has limited self-repair ability due to its avascular, alymphatic and aneural features. The combination of three-dimensional (3D) printing and tissue engineering provides an up-and-coming approach to address this issue. Here, we designed and fabricated a tri-layered (superficial layer (SL), middle layer (ML) and deep layer (DL)) stratified scaffold, inspired by the architecture of collagen fibers in native cartilage tissue. The scaffold was composed of 3D printed depth-dependent gradient poly(ε-caprolactone) (PCL) impregnated with methacrylated alginate (ALMA), and its morphological analysis and mechanical properties were tested. To prove the feasibility of the composite scaffolds for cartilage regeneration, the viability, proliferation, collagen deposition and chondrogenic differentiation of embedded rat bone marrow mesenchymal stem cells (BMSCs) in the scaffolds were assessed by Live/dead assay, CCK-8, DNA content, cell morphology, immunofluorescence and real-time reverse transcription polymerase chain reaction. BMSCs-loaded gradient PCL/ALMA scaffolds showed excellent cell survival, cell proliferation, cell morphology, collagen II deposition and hopeful chondrogenic differentiation compared with three individual-layer scaffolds. Hence, our study demonstrates the potential use of the gradient PCL/ALMA construct for enhanced cartilage tissue engineering.

## Introduction

Hyaline articular cartilage shows limited ability of self-repair and functional regeneration after injury or disease because of the absence of blood vessels, lymph and nerves [1]. For decades, researchers have been committed to repair articular cartilage using biological means. Traditional therapies, such as debridement, microfracture, mosaicplasty and autologous chondrocyte implantation, have ineffective long-term results [2]. As an ideal strategy, tissue engineering which combines cells, scaffolds, growth factors and other bioactive molecules to construct biomimetic biological structures, has the potential to achieve complete cartilage regeneration [3, 4]. 

For the selection of cell source, the utility of chondrocytes is limited by its rapid dedifferentiation and donor site morbidity [5]. It has been reported that mesenchymal stem cells (MSCs) from bone marrow are attractive as an alternative cell source for articular cartilage regeneration because of their multilineage differentiation and strong proliferation and self-renewal ability [6–8]. Further, successful cartilage tissue engineering desire a biocompatible scaffold that offers a 3D microenvironment conductive to cell and tissue ingrowth, and possesses appropriate biomechanical properties, interconnected pore and bioabsorbability [4, 9]. However, construct such a scaffold remains an enormous challenge to be urgently addressed. Because articular cartilage is an anisotropic tissue, its zonal structure plays a key role in tissue function. The collagen content and arrangement, stiffness and bearing strain of articular cartilage gradually change in gradient from superficial zone, middle zone to deep zone [10, 11]. Meanwhile, collagen fibers are parallel to the surface of the joint in the superficial zone, angular in the middle zone and perpendicular to the surface of the joint in the deep zone [12]. The emergence of 3D printing provides an opportunity to construct a customized, porous 3D scaffold with appropriate mechanical properties similar to cartilage extracellular matrix (ECM) [13, 14]. In recent years, the use of multi-layer scaffolds loaded with stem cells and cocktail medium containing chondrogenic-inducing components to provide a chondrogenic microenvironment to support cartilage tissue has been extensively studied [15–17]. Besides, previous studies have not clearly demonstrated that a defined pore size can be used as a baseline for the scaffold on cartilage regeneration [18–20]. Micro-pores promote cell adhesion while macro-pores facilitate the absorption of nutrients and the discharge of metabolic waste, bone marrow MSCs (BMSCs) seem to prefer the former by comparison [21]. Thus, scaffold design of gradient pore size and pore geometry can mimic the zonal structure of articular cartilage with similar biomechanical properties to some extent.

In recent years, the research progress in tissue engineering has enhanced the development of scaffold materials such as composites or blends. Alginate, collagen and other hydrogels have been printed as articular cartilage constructs, but with poor mechanical properties [22]. As an FDA-approved aliphatic polyester, poly(ε-caprolactone) (PCL) has attractive advantages of tunability, chemical diversity, excellent mechanical properties and phenotype maintenance, and has been proved to be used for repairing cartilage defects [23, 24]. However, PCL is often composited with other polymers to overcome its hydrophobicity [25, 26]. Hydrogels are highly absorbent, act as a buffer between the scaffold and the cartilage, and prevent cartilage degeneration [27]. It has been reported that alginate, a biocompatible polysaccharide extracted from seaweed, is widely used in cartilage regeneration, with excellent biocompatibility, injectability, and ability to support encapsulated cells differentiation, and can be quickly gelled by the addition of Ca^2+^ [14, 28, 29]. Fortunately, the composite of PCL and alginate without any modification have been reported in cartilage tissue engineering due to alginate’s hydrophilicity [14, 30]. Moreover, a new type of photosensitive hydrogel (methacrylated alginate (ALMA)) has been widely favored by scholars in recent years [31–33]. Its photocuring ability is enhanced by the introduction of methacrylic anhydride groups on the chain of alginate molecules. Compared with the traditional crosslinking of divalent ions (calcium ions etc.), the photocurable crosslinking has high portability and good homogeneity inside the hydrogel.

Here, the purpose of this study was to investigate the potential use of the gradient PCL/ALMA composite scaffolds for enhanced cartilage tissue engineering. The PCL scaffolds were 3D printed, modified by plasma surface treatment and performed by scanning electron microscopy. The ALMA was facilely synthesized and confirmed by hydrogen nuclear magnetic resonance (^1^HNMR) and Fourier transform infrared spectroscopy (FTIR). Moreover, we hypothesize and confirm that PCL-gradient scaffolds impregnated with BMSCs-loaded ALMA would mimic the zonal organization of articular cartilage, possess appropriate mechanical strength, and lead to higher cell viability, cell proliferation, collagen II deposition and chondrogenic gene expression.

## Materials and methods

**Figure 1.**
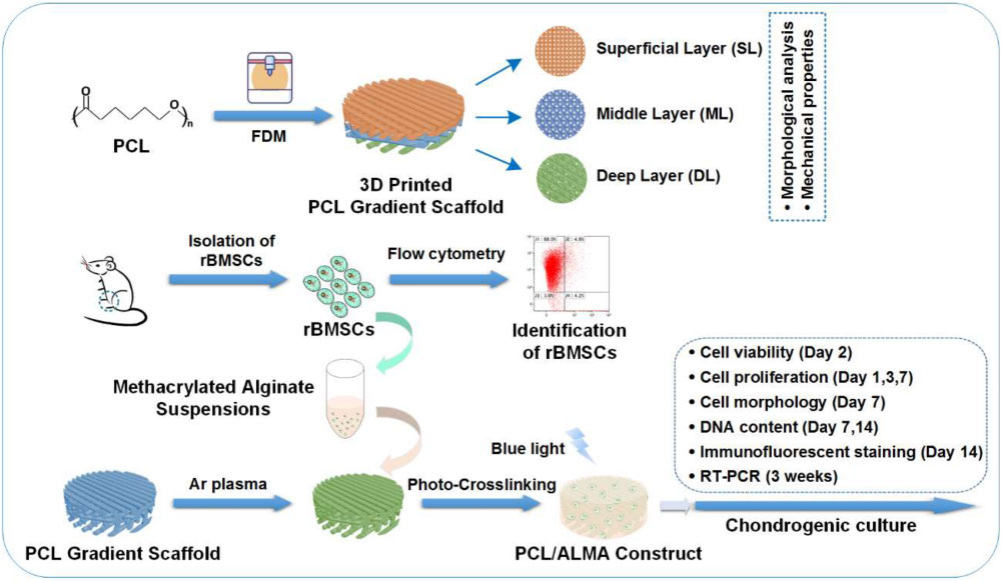
Flow diagram of the experimental process. FDM, fused deposition modeling; rBMSCs, rat bone marrow mesenchymal stem cells; ALMA, methacrylated alginate

A flow diagram is exhibited in Fig. 1 with all steps involved, in order to better outline the experimental process.

### Materials

In this study, PCL filament (molecular weight = 50 000 g/mol; 3D4Makers, Haarlem, The Netherlands) with a diameter of 1.75 mm and 4% (w/v) sodium alginate (Macklin, Shanghai, China) were used as a framework and hydrogel materials, respectively. Lithium phenyl-2,4,6-trimethylbenzoylphosphinate (LAP), *N*-(3-dimethylaminopropyl)-*N*-ethylcarbodiimide hydrochioride (EDC), *N*-hydroxysuccinimide (NHS), 2-morpholinoethanesulfonic acid (MES) buffer and 2-aminoethyl methacrylate hydrochloride were purchased from Sigma-Aldrich. All chemicals used were analytical grade.

### Design of the 3D PCL scaffolds

The PCL scaffolds were designed in a commercial computer aided design software (3ds Max, Autodesk). The final 3D model had a cylindrical geometry with dimensions of 14 mm (diameter) × 1.8 mm (height), 6 layers, 300 μm filament diameter, 300 μm layer thickness and different pore geometry (Fig. 3a). The superficial-layer (SL) architecture was defined as an orthogonal architecture with 0°/90° lay-down pattern and filament gap of 300 μm. There was offset between layers and the offset was set to half of the filament distance. Defined architectures were designed for middle-layer (ML) (filament gap of 500 μm, 0°/60° lay-down pattern) and deep-layer (DL) (filament gap of 700 μm, 0°/30° lay-down pattern). Three different lay-down patterns resulted in different pore structures, including rectangular, triangular and rhombic projection. Then, the upper two layers, middle layers and lower two layers of the gradient PCL scaffold were composed of SL, ML and DL, respectively.

### Synthesis of ALMA

ALMA was synthesized by reacting alginate with 2-aminoethyl methacrylate hydrochloride as previously described [32]. Briefly, the mixture of 1% w/v of alginate and 50 mM MES buffer was stirred until completely dissolved at pH 6.5 at room temperature. And 20 mM EDC, 10 mM NHS and 10 mM 2-aminoethyl methacrylate hydrochloride were added to the alginate solution, and the mixture was reacted for 24 h. The reaction was terminated by adding sufficient acetone. Then the precipitate was dissolved in phosphate buffered saline (PBS), dialyzed against deionized water (3.5 kDa MWCO) for 4 days to remove unreacted reagent, and filtered (0.22 μm filter). Finally, the resulting ALMA solution was lyophilized and stored at −20°C for further use.

### Characterization of ALMA

^1^HNMR was used to determine the degree of substitution of the modified ALMA. ALMA was dissolved in deuterium oxide (D_2_O) at a concentration of 50 mg/ml and transferred into nuclear magnetic resonance tubes after violent shaking. The spectra were recorded at a frequency of 500 MHz using a Bruker AVANCEIIIHD 500 NMR spectrometer.

The FTIR absorption spectra of ALMA and alginate were scanned with ThermoFisher Scientific^TM^ Nicolet^TM^ In^TM^ 10 by the potassium bromide (KBr) pellets method. The examination was performed at room temperature ranging from 400 to 4000 cm^−1^ with a resolution of 4 cm^−1^. Samples were lyophilized and ground into powder prior to measurement.

### Fabrication of the 3D printed PCL/ALMA composite scaffolds

The PCL scaffolds were printed by converting stereolithography files into g-code. PCL was melted and extruded through a 3D printer’s (SW100-3D, Taiyuan, China) heated metal nozzle, which was conducted by fused deposition modeling (FDM) software. The temperatures at the front and back of the printing head were 75°C and 125°C, respectively. The diameter of the nozzle was 400 μm, pneumatic pressure was 7 bar, printing speed was 20 mm/s and the control ratio of extrusion was 4%. After printing, the PCL scaffolds were allowed to cool down to room temperature. Then, they were removed from the glass plate and treated by radiation sterilization with 60 Co-γ rays.

To enhance hydrophilicity, the surface of PCL scaffolds was modified by mini plasma treatment system (Schwarze, P3C, Beijing Jiarun Power Technology Co., Ltd, China) in a vacuum chamber. All PCL scaffolds were exposed to Argon gas at a flow rate of 200  sccm, treatment time of 180 s and using power frequency at 40 kHz. Then, the changes in surface wettability of the plasma-treated PCL materials were evaluated by analyzing the contact angles using sessile drop method. Three replicates for each specimen were tested by using 1 μl of distilled water as medium on the digital microscope dino-light (AD4113T, Taiwan). Then the contact angles were analyzed by Dino Capture 2 software. The surface topography analysis of non-treated and Argon plasma treated 3D printed PCL scaffolds was performed by scanning electron microscopy (VEGA3 TESCAN, Czech Republic) operated at 10 kV under vacuum conditions.

Afterwards, they were rinsed in PBS three times, sterilized for 12 h under ultraviolet light, dipped in Dulbecco’s Modified Eagle’s Medium (DMEM) for 48 h prior to cell seeding. Besides, cells were suspended in solutions of 1.5% ALMA containing 0.3% w/v of the photoinitiator (PI, LAP, Sigma-Aldrich) in DMEM-F12 medium. Then, cells were first seeded onto a PCL scaffold for 4 h, the empty pores of the 3D printed PCL scaffolds were next filled with cells-loaded ALMA bioink (cell density: 3 × 10^5^ cells/ml, and then crosslinked by exposing to blue light (405 nm) for 30 s for polymerization). The PCL/ALMA composite scaffolds were cultured in DMEM/F12 supplemented with 10% fetal bovine serum (FBS), 100 U/ml penicillin and streptomycin in the 24-well plates.

### Morphological analysis of 3D PCL scaffolds

To evaluate the pore geometry, pore size and the physical integrity of the layers and filaments, morphological analysis of the 3D printed PCL scaffolds was performed by scanning electron microscopy (S4700 SEM, Hitachi Corporation, Tokyo, Japan) operated at 10 kV under vacuum conditions.

### Mechanical properties of PCL/ALMA composite scaffolds

To assess the effect of the pore size, pore geometry and lay-down pattern on the mechanical properties of 3D printed PCL/ALMA scaffolds, tensile tests and compressive tests were carried out with a mechanical test machine (Instron 3343) at 28 − 30°C [34]. Rectangular samples with a width of 10 mm and length of 40 mm and thickness of 3 mm were stretched with a speed of 30 mm/min at room temperature. Cylindrical samples (∼5 mm in diameter, 4 mm in height) were located on test plate and compressed with a speed of 0.3 mm/min. Vernier caliper was used to measure the length or thickness of specimens. The tensile and compressive moduli were calculated from the slope of the initial linear regions (5 − 20% strain) of the tensile and compressive stress strain curve, respectively.

### Isolation and identification of rat BMSCs

BMSCs were isolated from 6-week-old male Sprague-Dawley rat as described previously [35]. Care of the animals was in compliance with the Animal Ethics Committee of Shanxi Medical University. Briefly, the femora and tibiae were aseptically removed after euthanasia. The epiphyses were cut off and the marrow was flushed out using DMEM/F12 supplemented with 10% FBS (Gibco), 100 U/ml penicillin, 100 μg/ml streptomycin. After cultured for 2 days in an incubator (37°C, 5% CO_2_), the medium was replaced to remove non-adherent cells. BMSCs were cultured to 90% confluence, trypsinized with 0.25% trypsin/0.53 mM ethylenediaminetetra acetic acid (EDTA) (T1300, Solarbio, Beijing, China) and passaged at 1:2.

The specific cell surface antigen markers of rat BMSCs were detected by flow cytometry (FCM, Beckman Coulter, Navios). The expression of antibodies for positive markers included CD44 (Alexa Fluor 488, R&D systems), CD90 (FITC, BioLegend) and CD29 (APC, MACS), and the negative markers included CD45 (PE-Vio770, MACS) and CD11b (PE, BioLegend).

### Cell seeding and cells-scaffolds culture

At passage 3, 30 μl of rat BMSCs suspension with cell concentration of 3 × 10^5^ cells/ml was injected into the PCL scaffolds (SL-P, ML-P, DL-P, PCL-gradient) and then incubated for 20 min at 37°C and 5% CO_2_ atmosphere for initial adhesion. The exudation was assembled and reinjected into the scaffold to ensure cell seeding efficiency. Then, the PCL scaffolds were impregnated in BMSCs−ALMA suspensions (cell density: 3 × 10^5^ cells/ml) in the 24-well plates, and crosslinked by exposing to blue light (405 nm) for 30 s for polymerization to allow gelation. Thus, the composite PCL/ALMA cells-scaffolds (SL-PMA, ML-PMA, DL-PMA, PMA-gradient) were prepared.

The BMSCs-loaded PCL/ALMA scaffolds were cultured in growth medium (DMEM/F12 supplemented with 10% FBS and 1% penicillin and streptomycin). After 2 days, the BMSCs-loaded PCL/alginate scaffolds were re-cultured in chondrogenic differentiation medium (RASMX-90041, Cyagen, Suzhou, China), consisting of basal medium, 10 nmol/L dexamethasone, 50 ng/ml ascorbate, 1% insulin-transferrin-selenium (ITS + supplement), 100 μg/ml sodium pyruvate, 40 μg/ml proline and 10 ng/ml TGF-β3. The chondrogenic differentiation medium was changed twice a week.

### Cell viability

Cell viability in scaffolds was determined using Calcein-AM (Abbkine, Wuhan, China) and propidium iodide (PI, Solarbio) staining. The BMSCs-loaded PCL/ALMA composite scaffolds were cultured in growth medium for 48 h. Then the BMSCs-loaded scaffolds were rinsed twice with PBS, and each scaffold was submerged in 600 μl PBS with 2 μM Calcein-AM and 4 μM PI at 37°C for 2 h. The fluorescence of Calcein-AM (green in live cells) or PI (red in dead cells) was detected at excitation wavelength of 494 or 535 nm by a fluorescence microscope (Leica DMi8, Germany).

### Cell proliferation

The proliferation of rat BMSCs on scaffolds was determined on Day 1, 3 and 7 *in vitro* culture by a Cell Counting Kit-8 assay (CCK-8, Abbkine, Wuhan, China), following the manufacturers’ instructions. Briefly, the culture medium was discarded, the cell-seeded constructs (*n* = 3) were gently washed in PBS and then immersed with 500 μl of fresh DMEM/F12 supplemented with 10% FBS and 1% Penicillin/Streptomycin, containing 10% CCK-8 reagent at 37°C for 2.5 h. The supernatant was pipetted into 96-well plates in order to measure the absorbance at 450 nm by a microplate reader (Spectra Max M5, Molecular Devices, USA).

### DNA content

To assess the seeding efficiency of different types of constructs, dsDNA content was measured after 7 and 14 days of culture. PicoGreen assay (Quant-iT^TM^ PicoGreen^TM^ dsDNA Assay Kit, Invitrogen, Carlsbad, CA, USA) was implemented as previously mentioned [36]. All samples (*n* = 3) were digested in a papain buffer supplemented with 0.5 M EDTA, 0.05 M cysteine hydrochloride at 60°C for 12 h. A calf thymus DNA standard curve was applied to evaluate DNA content in each sample. The results were determined by a fluorescence microplate reader at an excitation wavelength of 480 nm and an emission wavelength of 520 nm.

### Cell morphology

Cell cytoskeleton experiment was performed with 4′,6-diamidino-2-phenylindole (DAPI, Boster, Wuhan, China) and Phalloidin (TraKine^TM^ F-actin Staining Kit, Abbkine, Wuhan, China) after 7 days of chondrogenic culture. The BMSCs-loaded constructs were fixed with ice-cold 4% formaldehyde for 30 min, permeabilized with 0.1% Triton X-100 in PBS for 10 min, incubated for 20 min in 1% bovine serum albumin in PBS, for 45 min in green fluorescence dye-labeled phalloidin to stain the f-actin and for 15 min in DAPI to stain the nuclei all at room temperature, and photographed using a confocal laser scanning microscope (CLSM, Leica TCS SP8, Germany).

### Immunofluorescent staining

After 2 weeks of chondrogenic culture, the immunofluorescent staining was performed to detect collagen II within different types of constructs. The cell-seeded constructs were fixed with 4% formaldehyde for 30 min and permeabilized with 0.1% Triton X-100 in PBS for 10 min. Then the samples were incubated for 1 h in 5% normal goat serum for blocking, in rabbit primary antibody against rat collagen II (BA0533, Boster) (dilution 1:200) at 4°C overnight. Next, the samples were incubated in FITC-labelled anti-rabbit IgG (BA1105, Boster) (dilution 1:32) for 1 h at room temperature, and for 15 min in DAPI. Photographs were taken with a CLSM, and the signal intensities of the CLSM images (*n* = 2 − 5 images/sample) were quantified with ImageJ.

### Real-time reverse transcription polymerase chain reaction

After 3 weeks of chondrogenic culture, total RNA was extracted using Trizol reagent (Invitrogen) as per the manufacturer’s instructions. The obtained white RNA pellet was dissolved in RNase-free water and the RNA concentration was measured with spectrophotometer (NanoDrop One^C^, Thermo Scientific). Complementary DNA (cDNA) produced by reverse transcription of isolated RNA using PrimeScript^TM^ RT Master Mix (TaKaRa), and the analysis of real-time reverse transcription polymerase chain reaction (RT-PCR) was carried out by Applied Biosystems^TM^ QuantStudio^TM^ 6 Flex RT-PCR System with TB Green^TM^ Premix Ex Taq^TM^ II (TaKaRa). The RT-PCR procedure was set as 95°C for 30 s, followed by 40 cycles of 95°C for 5 s, 60°C for 30 s, and then dissociation (95°C for 15 s, 60°C for 30 s and 95°C for 15 s).

The relative gene expression levels were calculated by the 2^-△△Ct^ method using PA903 as the reference group. Genetic markers related to chondrogenic differentiation were assessed, including Aggrecan, collagen II, collagen I, collagen X and SRY(sex determining region Y)-related high mobility group-box9 (SOX9). The expression levels of these target genes were investigated using glyceraldehyde-3-phosphate dehydrogenase (GAPDH) as a housekeeping gene for normalization. The PCR primer sequences are shown in Table 1.

**Table 1.** The primer sequences for RT-PCR

Gene	Forward primers (5’→3’)	Reverse (5’→3’)
Aggrecan	CTGATCCACTGTCCAAGCACCATG	ATCCACGCCAGGCTCCACTC
Collagen II	ACGCTCAAGTCGCTGAACAACC	ATCCAGTAGTCTCCGCTCTTCCAC
SOX9	TCAACGGCTCCAGCAAGAACAAG	CTCCGCCTCCTCCACGAAGG
Collagen I	GGGCAACAGCAGATTCACCTACAC	CAAGGAATGGCAGGCGAGATGG
Collagen X	GGATGCCTCTTGTCAGTGCTAACC	TCATAGTGCTGCTGCCTGTTGTAC
GAPDH	GCCACATCGCTCAGACACC	CCCAATACGACCAAATCCGT

### Statistical analysis

SPSS 22.0 software was applied for the statistical evaluation, and all of the data were expressed as mean ± standard deviation (SD). All results were repeated at least three times. A one-way analysis of variance (ANOVA) was used for comparing the differences between the groups. *P*<0.05 was regarded as the significant statistically difference. ‘NS’ shows no statistical significance.

## Results

### Synthesis and characterization of ALMA

**Figure 2.**
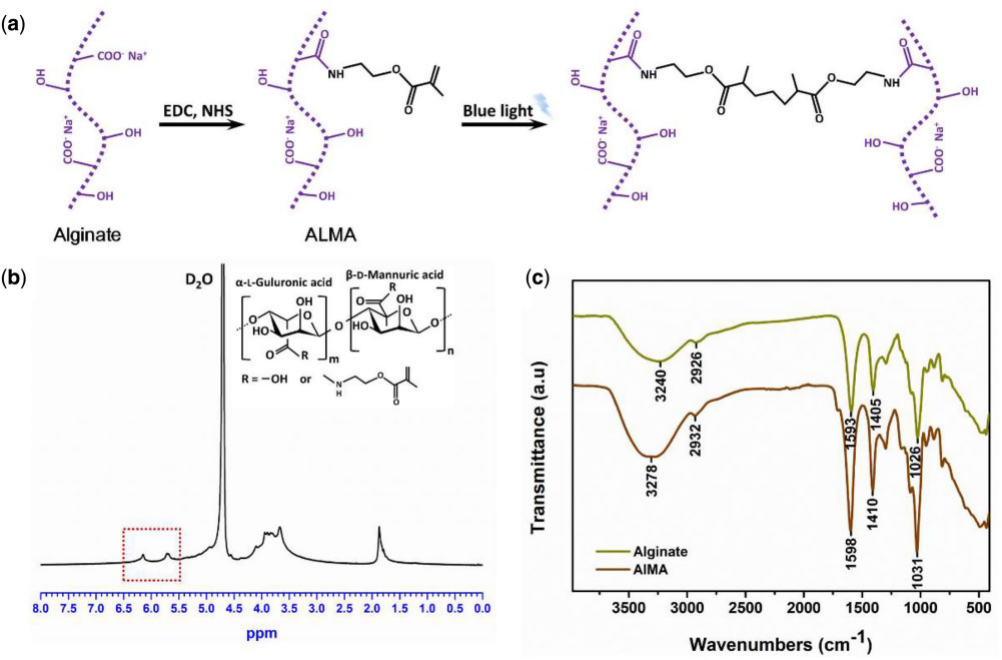
Synthesis and characterization of methacrylated alginate (ALMA). (**a**) Schematic illustration of preparation of ALMA by chemical modification of alginate. (**b**) ^1^HNMR spectra of synthesized ALMA. (**c**) The FTIR spectra of alginate and ALMA

Alginate is a promising polysaccharide, which is widely used in a variety of biomedical applications. In our study, ALMA is a photocrosslinked hydrogel derived from alginate. Alginate was methacrylated as described previously [32] and the chemical structure of ALMA was confirmed by ^1^HNMR and FTIR spectra (Fig. 2). The signals at 5.71 and 6.11 ppm represented vinyl and methyl protons, which was introduced by 2-aminoethyl methacrylate (Fig. 2b). The degree of functionalization was equivalent to about 20% according to the results of the ^1^HNMR. FTIR spectra of the alginate and ALMA were shown in Fig. 2c. The asymmetric and symmetric stretching peaks of carboxylate groups appeared near 1598 and 1410 cm^−1^, respectively [37]. The ALMA spectrum had a strong and broad hydroxyl peak at 3278 cm^−1^, and a C−O stretching vibration peak at 1031 cm^−1^. In particular, the new peak at 1717 cm^−1^ was featured by the C−O stretching of the methacrylic moiety. The results FTIR spectra further confirmed the synthesis of ALMA.

### Fabrication and characterization of the tri-layered PCL/ALMA constructs

**Figure 3.**
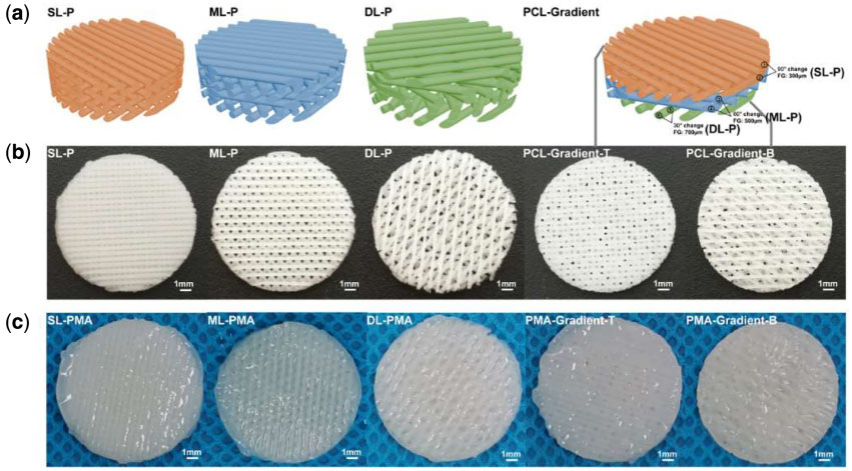
Models and gross morphology of scaffolds. (**a**) 3D models of PCL scaffolds, including SL-P, ML-P, DL-P and PCL-gradient. (**b**) Gross morphology of PCL scaffolds. (**c**) Gross morphology of PCL/ALMA scaffolds. PMA, PCL/ALMA scaffold; PCL-gradient-T, the top of the PCL-gradient scaffold; PCL-gradient-B, the bottom of the PCL-gradient scaffold; PMA-gradient, PCL-gradient scaffold impregnated with ALMA

**Figure 4.**
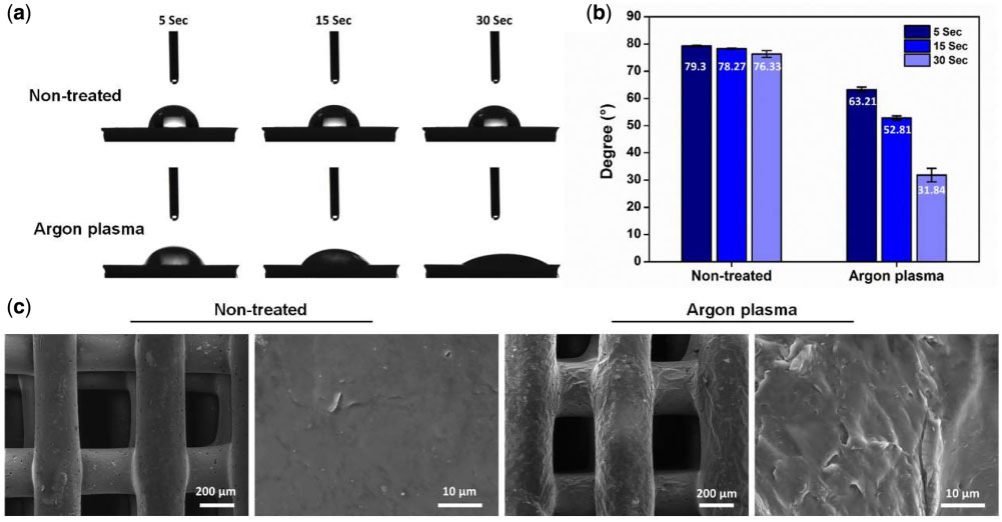
Comparison of (**a**) static water contact angle projections, (**b**) contact angle values and (**c**) SEM images of non-treated and argon plasma treated PCL scaffold

**Figure 5.**
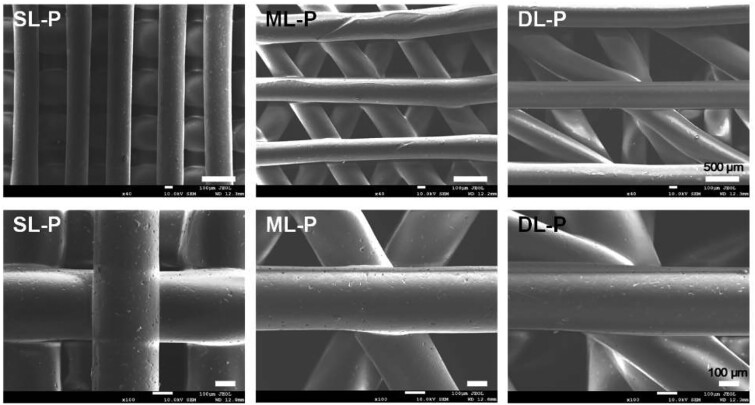
SEM Images of tri-layered PCL scaffolds. SL-P, superficial-layer, filament gap of 300 μm, 0°/90° lay-down pattern; ML-P, middle-layer, filament gap of 500 μm, 0°/60° lay-down pattern; DL-P, deep-layer, filament gap of 700 μm, 0°/30° lay-down pattern

In this study, the tri-layered PCL/ALMA constructs were performed through 3D printing, ALMA permeation and photocrosslinking as illustrated in Fig. 1. Digital photographs of PCL and PCL/ALMA scaffolds with 14 mm in diameter and ∼1.6 mm in height were shown in Fig. 3. As shown, ALMA exhibited a high degree of penetration into the pores of the scaffold. Meanwhile, after Argon plasma, contact angle of PCL surface can be reduced from 79.3° to 31.8° as shown in Fig. 4. This is because Argon can activate the surface of PCL fibers and include some functional group onto the surface, thus enhancing the hydrophilicity of PCL [38]. The surface morphology of the scaffolds exhibited controllable pore structures and uniform pore size. The SEM images further verified that 3D printed tri-layered PCL scaffolds had different filament gap, lay-down patterns (SL, filament gap of 300 μm, 0°/90° lay-down pattern; ML, filament gap of 500 μm, 0°/60° lay-down pattern; DL, filament gap of 700 μm, 0°/30° lay-down pattern), and homogeneously pore morphology with highly interconnected pores (Fig. 5). The high magnification images exhibited that all scaffolds possess clearly contoured inner pore architecture and good adhesion between neighboring layers. Quadrangular, triangular and complicated pore geometries with different filament gap and lay-down patterns were obtained. To confirm the precision, completeness, repeatability and stability of the 3D printing system, the filament diameter and layer thickness were measured using image J software. The actual measured filament diameter and layer thickness were in the range of 289.3 − 345.7, 266.7 − 281.2 μm, respectively. A remarkable agreement between the theoretical and actual values was achieved.

**Figure 6.**
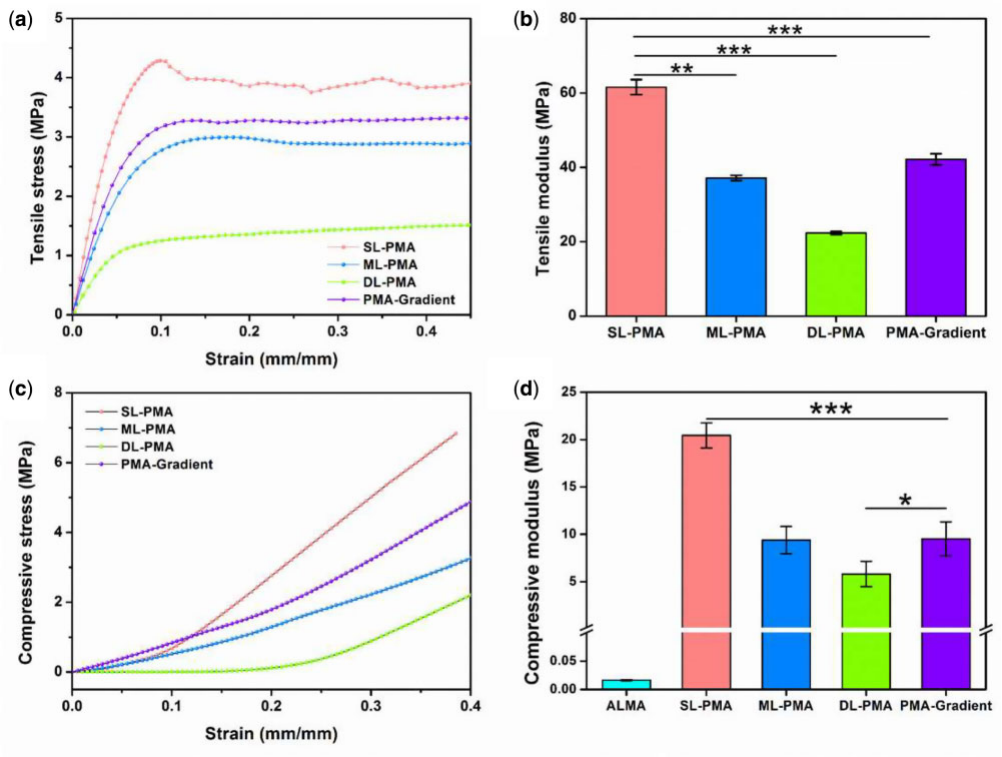
Tensile and compression test results of PCL/ALMA scaffolds. (**a**) Tensile stress−strain curves and (**b**) tensile moduli of PCL/ALMA scaffolds. (**c**) Compressive stress−strain curves and (**d**) compressive moduli of PCL/ALMA scaffolds. The superficial-layer, middle-layer, deep-layer networks and tri-layered networks are, respectively, represented as SL-PMA, ML-PMA, DL-PMA and PMA-gradient. ***P <* *0.001; **P <* *0.01; *P <* *0.5

Subsequently, we recorded the tensile and compressive response of the PCL/ALMA scaffolds with mono-layered and tri-layered networks (Fig. 6). The tensile and compressive modulus were derived from the slope of the initial linear area of the stress−strain curves. Compared with scaffolds with lager pore sizes, scaffolds with smaller pores sizes enjoyed higher stress [39, 40]. As depicted in Fig. 6, SL networks characterized by 0°/90° lay-down pattern exhibited the tensile modulus (61.57 ± 2.05 MPa) and compressive modulus (20.44 ± 1.32 MPa) which were greater than obtained other-layer networks characterized by other lay-down patterns. Castilho *et al.* [41] have shown that the superficial layer not only ensures the uniform distribution of compressive loads applied, but also stabilizes the collagen filament network in the middle and deep layer. Compared with pure ALMA (16.38 ± 1.09 kPa), PCL/ALMA composite scaffolds exhibited higher bearing capacities. In terms of four kinds of networks including SL-PMA, ML-PMA, DL-PMA and PMA-gradient, the trend of tensile and the compressive modulus were SL-PMA > PMA-gradient > ML-PMA > DL-PMA. Both the tensile and compressive modulus decreased significantly with the increase of cartilage depth. The result demonstrated that PMA-gradient scaffold was closer to ML-PMA due to the average spacing of filaments. The compressive modulus of PMA-gradient scaffold was 9.52 ± 1.79 MPa, which were close to the mechanical strength of natural cartilage tissue and met the mechanical properties of engineered cartilage tissue [42, 43]. In this regard, we considered that PMA-gradient scaffold had more suitable mechanical properties.

### Identification of rat BMSCs

**Figure 7.**
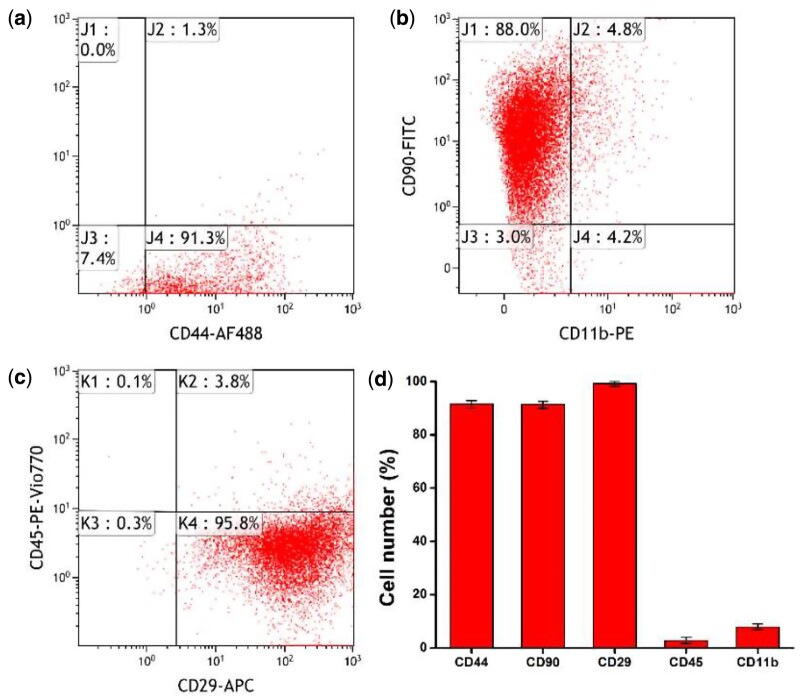
Scatterplots for identification of rat BMSCs via flow cytometry. The expressions of (**a**) CD44, (**b**) CD90 and CD11b, (**c**) CD29 and CD45 were detected in 92.6, 92.8% and 9, 99.6 and 3.9% of the cells, respectively. (**d**) The histogram of cell number. Columns represent mean values and error bars represent SD

After isolation and amplification, the characterizations of rat BMSCs were examined at Passage 3, and the cells started to exhibit homogeneous phenotypes. FCM analysis indicated that the rat BMSCs included positive expression for CD44 (91.53 ± 1.36%), CD90 (91.33 ± 1.27%) and CD29 (99.17 ± 0.93%). In contrast, other markers of the hematopoietic lineage were expressed negatively, including the leukocyte common antigen CD45 (2.77 ± 1.21%) and CD11b (7.87 ± 1.10%) (Fig. 7). The results confirmed the purification of rat BMSCs.

### Cell viability

**Figure 8.**
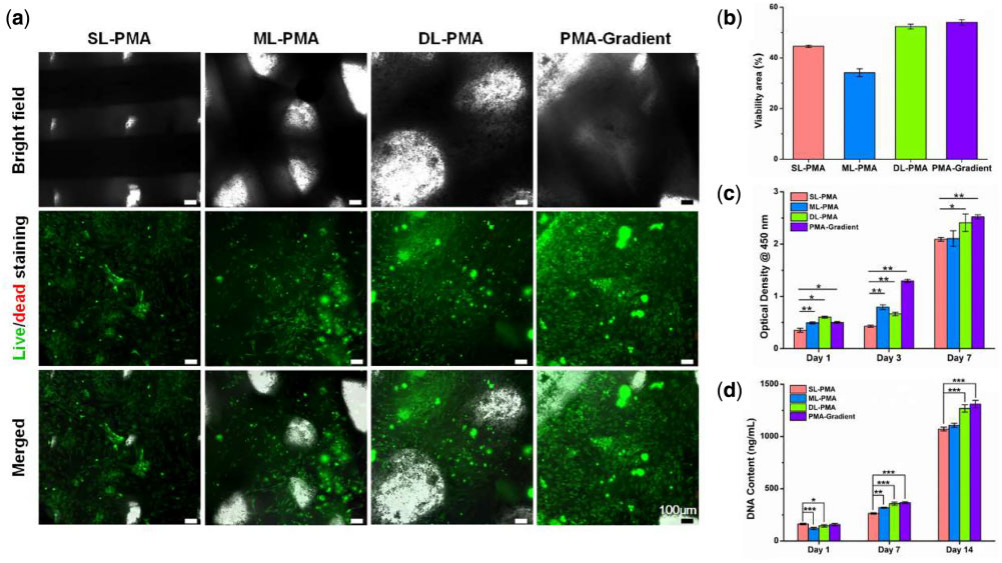
*In vitro* culture of rat BMSCs loaded on PCL/ALMA composite scaffolds. (**a**) The viability of rat BMSCs *in vitro* cultured on PCL/ALMA composite scaffolds. Bright field images showing the contours of the composite scaffolds. Live/dead staining indicates cell activity of three individual zonal scaffolds (SL-PMA, ML-PMA and DL-PMA) and PMA-gradient composite scaffold after culture for 48 h *in vitro* (green, living cells labeled with Calcein-AM; red, dead cells labeled with PI). The distribution of rat BMSCs on PCL/ALMA composite scaffolds was shown in the merged images. Scale bars represent 100 μm. (**b**) Quantification of cell viability. Cell proliferation analysis of 3D printed PCL/ALMA composite scaffolds. (**c**) CCK-8 assay showing a measurable increase of rat BMSCs in four groups after 1, 3 and 7 days. (**d**) A similar trend is also shown in DNA content after 1, 7 and 14 days

The live/dead assay was performed and the living and dead cells were quantified after culture in growth medium for 48 h to estimate cell viability. It was apparent from Fig. 8a that rat BMSCs survived well in PCL/ALMA composite scaffolds with minimal dead cells. The rat BMSCs embedded in composite scaffold exhibited a uniform distribution with polygonal and lanky appearance, and filled in pores. These results suggested that PCL/ALMA hybrid scaffolds had excellent compatibility with rat BMSCs. Moreover, compared with three individual zonal scaffolds (SL-PMA, ML-PMA and DL-PMA), PMA-gradient composite scaffold swarmed with ALMA seemingly furnished an appropriate cartilage-growth microenvironment, which was confirmed by the formation of some larger cell masses and homogeneous distribution.

### Cell proliferation

CCK-8 assay showed that rat BMSCs cultured in PCL/ALMA scaffolds have a measurable increase *in vitro* culture for 7 days (Fig. 8c). Nevertheless, the quantity of cells in SL-PMA, ML-PMA and DL-PMA groups on Day 3 did not increase markedly compared with Day 1. By contrast, rat BMSCs cultured in PMA-gradient scaffold exhibited a significant increase and outstripped that in the other individual zonal scaffolds at 7 days. The result could be correlated to the fact that gradient composite scaffolds presented higher porosity and specific pore structure, which offer more accessible sites for cells.

DNA contents of PCL/ALMA scaffolds were assessed at 1, 7 and 14 days after seeding (Fig. 8d). Compared with the first 7 days, DNA contents in all groups had increased about 3-fold at 14 days, which were in line with the growth trend measured by CCK-8. It was worth mentioning that the results were similar between the two groups of DL-PMA and PMA-gradient. Moreover, DNA contents of PMA-gradient group achieved a higher level and also surpassed other groups at 14 days.

### Cell morphology and collagen deposition assessments

**Figure 9.**
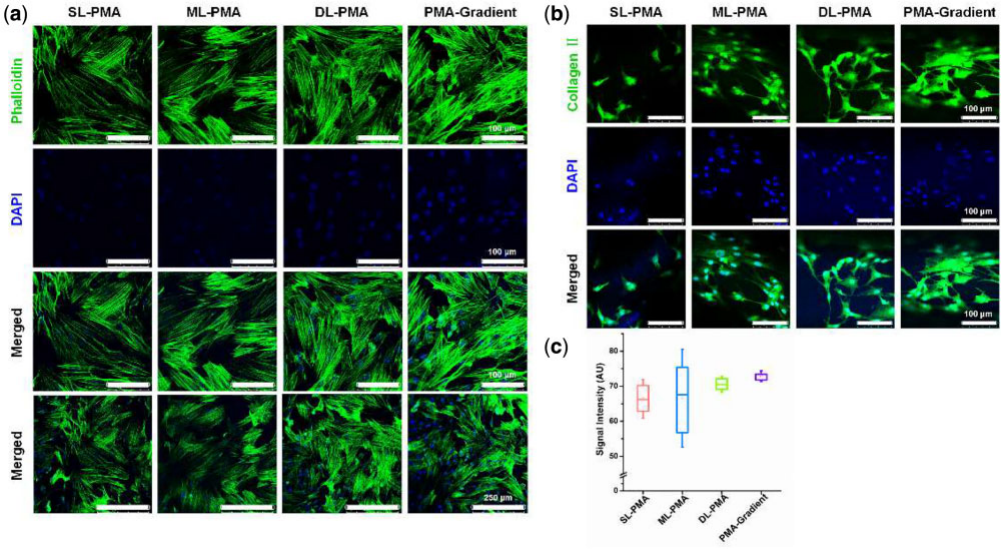
Cell morphology and collagen II deposition on PCL/ALMA constructs after chondrogenic incubation. (**a**) Confocal laser scanning microscopy (CLSM) images showing cell morphology on the PCL/ALMA scaffolds after 7 days of chondrogenic incubation. Nuclei: blue (DAPI), and f-actin: green (green fluorescence dye-labeled phalloidin). (**b**) CLSM images of immunostaining showing deposition of collagen II on the constructs after 2 weeks of chondrogenic incubation. Nuclei: blue (DAPI), and collagen II: green (FITC-labelled antibody). (**c**) Quantitative analysis of the CLSM images showing the intensity of collagen II signal intensities

We examined the effect of scaffold structure on cell morphology. After chondrogenic culturing for 7 days, BMSCs were elongated and aligned along the PCL strands, but randomly distributed without a certain pattern on ALMA (Fig. 9a). The cells in PMA-gradient scaffolds showed obvious adhesion morphology and had the greatest cell area. Immunostaining of PCL/ALMA scaffolds revealed low collagen deposition on SL-PMA and ML-PMA, and high deposition on DL-PMA and PMA-gradient (Fig. 9b). Quantitative analysis revealed the highest intensity of collagen II was on PMA-gradient scaffold (Fig. 9c).

### Chondrogenic-specific genes expression

**Figure 10.**
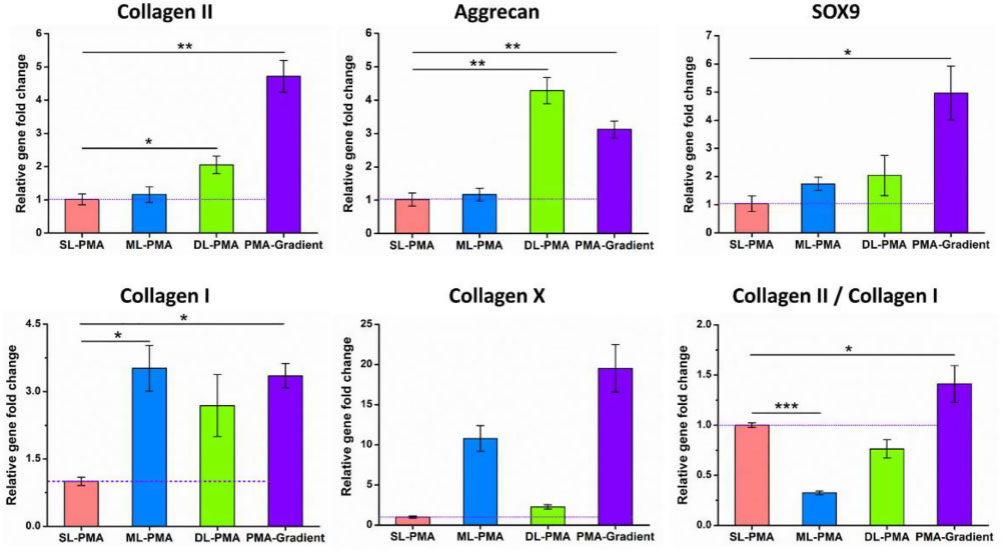
RT-PCR Results for chondrogenic-specific genes expression level of collagen II, aggrecan, SOX9, collagen I and collagen X of rat BMSCs cultured in various scaffolds after 3 weeks of chondrogenic differentiation *in vitro*. The values are given as fold change and normalized to GAPDH value (*n* = 3). The dotted purple line shows the gene expression level of the control group (SL-PMA)

After 3 weeks of chondrogenic differentiation of rat BMSCs in PCL/ALMA composite scaffolds of SL-PMA, ML-PMA, DL-PMA and PMA-gradient were analyzed by RT-PCR. The gene expressions of collagen II, aggrecan, SOX9, collagen I and collagen X were measured to compare the chondrogenic capacity (Fig. 10). According to the figure, greater upregulated expression of chondrogenic-specific genes of collagen II, aggrecan and SOX9 were observed in all four groups of composite constructs. Collagen II is mainly produced by chondrocytes, aggrecan is mainly distributed in the ECM, and SOX9 is the earliest markers for MSC differentiation into chondrocytes [44]. Collagen X is commonly associated with chondrocyte hypertrophy and is expressed in MSCs that undergo chondrogenesis after 21 days of culture [45]. Moreover, the PMA-gradient scaffold exhibited significantly higher expression levels of collagen II, collagen X and SOX9 in comparison to the other networks, and the expression of aggrecan was slightly lower than DL-PMA networks. Besides, osteogenesis-related collagen I was the lowest on SL-PMA, and there was little difference in the other three groups. However, collagen II/collagen I ratio (the chondrogenic differentiation index) was higher on SL-PMA, but highest on PMA-gradient. These results were assumed that PMA-gradient scaffold could provide a better microenvironment for chondrogenic differentiation of rat BMSCs.

## Discussion

Articular cartilage possesses a higher anisotropy and an internal zonal structure [46], and 3D printing allows preparation process of tissue engineering scaffold to be precise and controllable. The collagen fibers are arranged neatly parallel to the surface of the cartilage in the superficial zone, scattered and distributed at a certain angle to the cartilage surface in the middle zone, and perpendicular to the articular surface in the deep zone [10, 11, 47]. Scaffolds that mimic the structure of articular cartilage could induce ECM production in different regions and further lead to long-term cartilage regeneration. Hence, we paid more attention to design and produce a PCL/ALMA gradient composite scaffold (PMA-gradient) mimicking the zonal organization in normal cartilage. This was done by 3D printing a PCL scaffold with fully interconnected pores and gradient pore geometry along the longitudinal direction and impregnating with ALMA hydrogel (Fig. 3). This precise control of scaffold design allowed a direct comparison of the cellular and ECM production responses to the scaffold structure. The superficial, middle and deep zones of PMA-gradient scaffolds were composed of SL-PMA, ML-PMA and DL-PMA, respectively. By comparing the morphology, mechanical and biological properties of each part, the applicability of PMA-gradient composite scaffolds for cartilage regeneration was evaluated.

It is well known that pore geometry of the scaffold is important and helpful for cell survival, ingrowth and proliferation [20]. Many researchers have proposed scaffolds based on 3D printed PCL for articular cartilage regeneration, but most of them adopted 0°/90° lay-down pattern and did not intend to mimic the zonal biochemical components of articular cartilage [14, 23, 25, 30]. From our study, SEM photographs of three kinds of PCL scaffolds with different lay-down pattern and filament gap were shown in Fig. 5. The theoretical values of filament diameter and filament gap were remarkable consistent with the experimental values, which verify the accuracy of 3D printing. Comparison of the findings with those of other studies confirmed that all the PCL scaffolds fabricated by 3D printing had interconnected pores with customized filament gap of 300 − 700 μm and deposition angles between the neighboring layers of 30°−90°, all favorable for cell interactions [39, 40]. In order to mimic the zonal structure of articular cartilage, PMA-gradient scaffold was designed. And three kinds of networks including SL-PMA, ML-PMA, DL-PMA were chosen as control groups for emphatic research and comparison with PMA-gradient scaffold in mechanical properties (Fig. 6) and cell experiments (Figs 8 − 10).

In terms of cartilage tissue engineering, especially for weight-bearing joints, the graft should have sufficient mechanical strength to withstand stress of surrounding tissue. The compressive tests have always been carried out to evaluate the biomechanical properties of cartilage, while tensile tests were rarely applied and often overlooked. Actually, tensile strains were inherent in articular cartilage, and one of major metrics for mechanical properties of cartilage tissue [48, 49]. Moreover, existing study showed that tensile forces had an active influence on the differentiation of MSCs [50]. Thus, the PMA-gradient scaffold with three-part of pore structure could average mechanical characteristics of each part of scaffolds of SL-PMA, ML-PMA and DL-PMA, and obtained the tensile modulus of 42.14 ± 1.53 MPa and compressive modulus of 9.52 ± 1.79 MPa as shown in Fig. 6. It is noteworthy that the compressive moduli of scaffold in three layers reached a level comparable to that of native cartilage (2.75–21.4 MPa) [51]. Moreover, the tensile modulus of PMA-gradient scaffold (42.14 ± 1.53 MPa) was obviously comparable to that of human articular cartilage (8.65–91.09 MPa) [51, 52]. Prior studies have noted that the PCL scaffolds impregnated with hydrogel had a slightly higher compressive modulus due to its higher water content and elasticity [53]. Consequently, the construction of PMA-gradient scaffold could provide essential physical and mechanical support for cellular growth, and obtain suitable compressive modulus under the infiltration of hydrogel.

In addition, functional scaffold with appropriate surface for cell adhesion and proliferation is expected. Hydrogels are hydrophilic, facilitate the absorption of nutrients and the discharge of metabolic waste, and have been used as scaffolds for cartilage regeneration [54]. Alginate is a naturally derived polysaccharide with excellent biocompatibility, gelation and ability to support cell differentiation [55, 56]. Herein, we not only modified the surface of PCL scaffolds with plasma treatment system, but also impregnated with photocrosslinking ALMA to further enhance hydrophilicity. The presence of ALMA within the pores of PCL scaffold can satisfy the requirements of cartilage tissue engineering for implant materials. As shown in Fig. 8a, the PMA-gradient scaffold offered a favorable growth micro-environment for cellular distribution and differentiation, which was proved by live/dead assay after cultured 48 h. The wrapped rat BMSCs were uniformly distributed into ALMA filled the interconnected pores and aggregated on the surface of scaffold. All independent scaffolds of SL-PMA, ML-PMA and DL-PMA also exhibited good cellular activity. The trend of cell viability was DL-PMA > SL-PMA > ML-PMA. Cell viability was strongest in the deep layer of the PCL/ALMA scaffold (Fig. 8b), consistent with that of native cartilage [47]. Previous work from others have demonstrated the hydrophobicity of PCL [24, 25, 30], therefore, it is necessary to note that PCL scaffolds were not used for cell experiments in our study.

The cell proliferation in tri-layered PCL/ALMA scaffolds were revealed by CCK-8 assay, whose results were also consistent with the findings of DNA content (Fig. 8c and d). The cells appeared to grow faster in the deep zone scaffolds (DL-PMA) and PMA-gradient compared with that in superficial zone scaffolds (SL-PMA) and middle zone scaffolds (ML-PMA). This may be attributed to the larger pore size of scaffold allowing more hydrogel to be attached, which is conductive to transport of oxygen and nutrients, as well as cell survival and proliferation. The present work demonstrated that biodegradable PMA-gradient scaffold was suitable for the efficient expansion of rat BMSCs.

Besides, we investigated the effects of PCL/ALMA scaffolds on cell morphology and collagen deposition (Fig. 9). Results clearly showed that PMA-gradient scaffold induced cell elongation and alignment. The cells in each layer of the scaffold showed obvious adhesion morphology, and cells in deep layer had the greatest cell area. Because arrangement of cells plays an important role in promoting the secretion of well-arranged collagen, PMA-gradient scaffold could mimic the zonal organization in the longitudinal direction of native cartilage. PCL could force cells to elongate, while ALMA could cause low cell diffusion and rounded cell morphology, and lead to chondrogenic phenotype. The most intense collagen II immunofluorescence staining was PMA-gradient (Fig. 9c). Since PCL exhibited low levels of collagen II [57], introduction of ALMA into PCL scaffolds significantly enhanced collagen II production.

To assess the chondrogenic capacity of encapsulated rat BMSCs in PCL/ALMA scaffolds, collagen II, aggrecan, SOX9, collagen I and collagen X gene expression were found after 3 weeks *in vitro* chondrogenic culture (Fig. 10). The loaded rat BMSCs in PMA-gradient scaffold showed a similarly robust higher level of gene expression of collagen II, collagen X and SOX9 compared with that on the three part scaffolds. SOX9 binds to the enhancer sequence of collagen II and is of great significance to the early stage of cartilage formation [58]. Interestingly, rat BMSCs loaded on the deep zone scaffold (DL-PMA) exhibited a slight stronger aggrecan expression compared with that in PMA-gradient scaffold. Moreover, compared with SL-PMA, greater upregulated expression of chondrogenic-specific genes of collagen II, aggrecan and SOX9 were observed in both ML-PMA and DL-PMA of the composite constructs. The gene expressions of collagen II and aggrecan in DL-PMA were the highest. Besides, collagen X and osteogenesis-related collagen I were the lowest in SL-PMA, while collagen II/collagen I ratio (the chondrogenic differentiation index) was higher, which was consistent with the study by Nguyen *et al*. [59].

Based on previous findings, the use of biocompatible hydrogels in cartilage defects usually fails to achieve satisfactory mechanical strength and load-bearing capacity [60]. In order to better simulate the structure and function of cartilage, hydrogels were combined with fibrous scaffolds to construct fibrous reinforced scaffolds [12, 41]. Researchers have shown that, in addition to serving as mechanical reinforcement, fiber orientation can provide structural and biochemical cues that regulate cells behavior to induce specific cell adhesion, growth and differentiation [61]. Moreover, the use of multi-layer scaffolds loaded with stem cells to provide a chondrogenic microenvironment to support cartilage tissue has been extensively studied [15]. To determine the ability of the multi-layer scaffold to support cartilage tissue formation, BMSCs were incorporated into the respective layers and cultured in chondrogenic medium. In this study, the tri-layered PCL/ALMA scaffold realized horizontal arrangement of cells in the top layer, and angular or vertical arrangement of cells in the middle or deep layer. SL-PMA mimicked the superficial layer of cartilage to ensure smooth surface lubrication and uniform distribution of applied loads. The cartilage-specific genes of collagen II and aggrecan were the highest in DL-PMA. Despite these interesting findings, the present study still has limitations. The fibrous pattern of each layer of the scaffold cannot accurately simulate the fibrous structure of natural cartilage tissue, and further efforts are needed. Subsequent studies should also take in consideration the repair of osteochondral defects, including the simulation of natural calcified cartilage and subchondral bone.

In a word, we have made a preliminary study and mainly concentrated *in vitro* applicability evaluation of PCL/ALMA composite scaffolds, and we believe PMA-gradient could be a candidate scaffold for cartilage regeneration and are very hopeful for future studies *in vivo*.

## Conclusions

In this study, we demonstrated a novel composite construct consist of a 3D printed tri-layered PCL scaffolds impregnated with ALMA, which had fully interconnected pores and gradient pore geometry along the longitudinal direction. As verified by scaffold structure, tensile and compressive test, and *in vitro* cell responses, the PMA-gradient scaffolds were more conductive to the attachment, proliferation and chondrogenic differentiation of rat BMSCs. These findings revealed the fact that gradient PCL/ALMA composite scaffold mimicking the zonal organization in native cartilage could provide a favorable microenvironment for enhanced cartilage tissue engineering. Based on these results, co-printing of PCL and hydrogels and future studies *in vivo* are recommended.

## Ethical approval

In this study, all methods were carried out in accordance with relevant guidelines and regulations. All animal experiments were approved by the Animal Ethics Committee of Shanxi Medical University. All procedures on animals were in compliance with the animal protection agreements and regulations.
